# Supplementary material for: Creatine kinase is associated with glycated haemoglobin in a nondiabetic population. The Tromsø study
Source: PLoS One. 2023 Feb 2;18(2):e0281239. doi: 10.1371/journal.pone.0281239 (PMC9894408; doi:10.1371/journal.pone.0281239)
Supplement: Attachment — Submitted filename: Response to Reviewers.docx [file pone.0281239.s001.docx]

Svein Ivar Bekkelund

Department of Clinical Medicine, UiT –

The Arctic University of Norway, Tromsø, Norway

and

Department of Neurology,

University Hospital of North Norway, Tromsø, Norway

Tromsø, July 29, 2022

Editor, PLoS ONE

PONE-D-21-32482R1

“Creatine kinase is associated with glycated haemoglobin in a nondiabetic population. The Tromsø study”.

Thank you for reviewing the manuscript and for considering it for publication in PLoS ONE. The responses to reviewers are carefully considered and described point by point below.

Reviewer #1: Manuscript #: PONE-D-21-32482
Title: Creatine kinase is associated with glycated haemoglobin in a nondiabetic population. The Tromsø study

CK is mainly influenced by the degree of physical activity, the type and number of drugs, and genetic factors. All these variables were not included in the present study. The influence of the Hba1c on CK can be only reliably assessed if the cohort had been homogenized for these influences

Reply:

Physical activity has been included in the variable list (Table 1) and adjusted for in the multiple variance analysis (Table 4). The main finding of the study; an independent association between glycated haemoglobin and creatin kinase, remained unchanged after adjusting for physical exercise. Definition of “heavy exerciser” is described in the methods.

Reviewer #2: The study examined the associations between CK and other CV risk factors in a large sample. It was found that CK was associated with these factors. These associations, however, were cross-sectional and important factors, such as physical activity, were not considered.

Reply: See above.

1) As this is a cross-sectional study, causal relationships cannot be established.

Reply: See below

2) As the authors discussed, an important factor influencing CK concentrations is physical activity, which was lacking in the study.
3) Systolic blood pressure is closed correlated to diastolic blood pressure. Including these two in the same model might have resulted colinearity. It would be advisable to use separate models for blood pressure.

Reply:

The multivariate analyses (Tables 3 and 4) are recalculated by including systolic blood pressure (model 1) and diastolic blood pressure (model 2) separately. These two models are presented side by side in the tables and explained in “Materials and methods” (statistical analyses).

4) According to the authors, the reference values for CK were age-dependent for men, but not for women (ref 21). Could the authors explain why?
5) Language in the manuscript needs further clean-up.

Reply:

I have reviewed the manuscript and tried to improve the language. Especially, the last paragraph in the discussion and the conclusion are modified. Limitations to the study and comments upon sex-differences are also included:

“An important limitation to the study is lack of causal information from the analyses of the HbA_1C_ vs. CK relationship due to the cross-sectional design. Possible influence of confounding factors is therefore important to be aware of. Muscle fibre 2B preponderance indicating impaired insulin sensitivity is associated with increased BMI [14, 29]. Moreover, higher frequencies of muscle fibre type 2B seem to increase with age, supporting the view that age may confound the association between HbA_1C_ and CK (Table 2) [30]. Hypothetically, muscle fibre morphology may therefore explain why CK associates with HbA_1C_. Age and BMI appear as confounders since both were positively and independently associated with HbA_1C_ and CK in the present study. Similar relationships between age, BMI and CK are reported by others [10, 12, 31] but less is known about the role of HbA_1C_. This also include sex-differences in this relationship as CK associated linearly with HbA_1C_ in women but not in men”.

Best regards,

Svein Ivar Bekkelund

Corresponding author
