# Supplementary material for: Creatine kinase is associated with glycated haemoglobin in a nondiabetic population. The Tromsø study
Source: PLoS One. 2023 Feb 2;18(2):e0281239. doi: 10.1371/journal.pone.0281239 (PMC9894408; doi:10.1371/journal.pone.0281239)
Supplement: Attachment — Submitted filename: Comments.docx [file pone.0281239.s002.docx]

This study aims to determine the association between creatine kinase and glycated haemoglobin (HbA1C) in a nondiabetic population. The authors found that creatine kinase (CK) was significantly and positively correlated with HbA1C in nondiabetic participants of the Tromsø Study. The sample size was large with a sufficient statistical power, and the finding is of interest. However, there are problems and concerns that need be addressed as below.

The major concern is that it is confusing throughout the paper regarding which one, HbA1c or CK, was used as the outcome variable.

In the Abstract, “Endpoint variables were measured cross-sectionally in 11662 nondiabetic subjects defined as HbA1C (%) <6.5 participating in the population-based Tromsø study (Tromsø 6) in Norway.” This statement is rather vague. It is not clear which variable was used as the outcome. “Additionally, HbA1C (%) was an independent predictor of high CK” indicated that CK is the outcome variable.

The conclusion “These data confirm a relationship between CK and glycosylation and conforms with the knowledge of the role of CK as a cardiovascular risk marker.” is not appropriate and needs to be revised. The data do not support “the role of CK as a cardiovascular risk marker”.

In Table 3, HbA1c was the dependent variable, but CK was the dependent variable in Table 4.

Diabetes was defined as HbA1c ≥6.5%. Please justify why glucose and anti-diabetic medication were not used for diagnosis.

Systolic and diastolic blood pressure should not be included in the same model. Their association parameters in Tables 3 and 4 are hard to explain due to the collinearity. Inclusion of systolic blood pressure is good enough.
