# Supplementary material for: Creatine kinase is associated with glycated haemoglobin in a nondiabetic population. The Tromsø study
Source: PLoS One. 2023 Feb 2;18(2):e0281239. doi: 10.1371/journal.pone.0281239 (PMC9894408; doi:10.1371/journal.pone.0281239)
Supplement: Attachment — Submitted filename: Response to reviewers.docx [file pone.0281239.s003.docx]

Svein Ivar Bekkelund

Department of Clinical Medicine, UiT –

The Arctic University of Norway, Tromsø, Norway

and

Department of Neurology,

University Hospital of North Norway, Tromsø, Norway

Tromsø, November 22, 2022

Editor, PLoS ONE

PONE-D-21-32482R1

“Creatine kinase is associated with glycated haemoglobin in a nondiabetic population. The Tromsø study”.

The responses to reviewers 1 and 3 and from the Editor are carefully considered and described point by point below.

Reviewer 1

The result and the main message of the study have not significantly changed. Other factors determining CK were not sufficiently included in the evaluation.

Reply:

Alcohol consumption, alanine transaminase, aspartate aminotransferase and gamma glutamyl transpeptidase are included, in Table 1 and those which were statically significantly associated with CK were used in the further analyses. The method of recruiting confounders is described in “statistical analyses”:

*Confounding variables identified by comparisons between groups with high CK and normal CK and by variance analyses were used to model the association between CK and HbA_1c_ (%). By multiple regression analysis, confounders with statistically significant values from these analyses were tested and adjusted for in relation to HbA_1c_ (%) as the dependent variable and log CK, age, BMI, systolic blood pressure, glucose, HDL- and LDL cholesterol, log hs-CRP, creatinine, ALT, and AST as independent variables. The data were reanalyzed in 9644 subjects after excluding those who reported* m*oderate* or hard *leisure physical* exercise ≥2 hours per week *(n=2018) and secondly after excluding1693 lipid lowering drug users (n=7951).*

A discussion of CK-related confounders is included at the end of the discussion:

*Physical activity is an obvious confounder in such a study. Despite reanalysing the data after excluding heavy physical exercisers, CK remained an independent predictor of HbA_1C_ (%). The impact of leisure physical activity on the CK-values in this population is previously estimated to be about 5% [8]. Likewise, a positive association between BMI, blood pressure and CK and an inverse relationship between CK and CRP are also previously reported [11, 20]. In line with findings from others, this study did not show higher CK in statin users [10]. Neither is ethnicity expected to play a major role since Caucasian populations like the present one is associated with lower CK levels than black- or mixed ethnic populations [10, 12], Knowledge about confounding variables related to CK may therefore be regarded as a strength of this study.*

Reviewer 3

The major concern is that it is confusing throughout the paper regarding which one, HbA1c or CK, was used as the outcome variable.

Reply:

The article is reviewed and the outcome variable HbA_1C_ and CK as the independent variable are made clear.

In the Abstract, “Endpoint variables were measured cross-sectionally in 11662 nondiabetic subjects defined as HbA1C (%) <6.5 participating in the population-based Tromsø study (Tromsø 6) in Norway.” This statement is rather vague. It is not clear which variable was used as the outcome. “Additionally, HbA1C (%) was an independent predictor of high CK” indicated that CK is the outcome variable.

Reply:

The method part in the abstract is changed to:

*Associations between CK and the outcome variable HbA_1C_ (%) were performed by variance and multivariate analyses in 11662 nondiabetic subjects defined as HbA_1C_ (%)* *<6.5 who participated in the population based Tromsø study (Tromsø 6) in Norway.*

The conclusion “These data confirm a relationship between CK and glycosylation and conforms with the knowledge of the role of CK as a cardiovascular risk marker.” is not appropriate and needs to be revised. The data do not support “the role of CK as a cardiovascular risk marker”.

Reply:

The conclusion is changed to:

*These data demonstrate a positive and independent association between CK and glycated haemoglobin in a nondiabetic general population.*

In Table 3, HbA1c was the dependent variable, but CK was the dependent variable in Table 4.

Reply:

Table 4 is deleted. Table 3 is now divided into models 1 (total sample) and 2 (total sample excluding heavy exercisers). Exercise as a confounder is commented upon in the last part of the discussion as referred to above (response to reviewer 1).

Accordingly, the background paragraph is changed to:

*..This study determined whether CK is a predictor of glycated haemoglobin (HbA_1C_) in a nondiabetic general population.*

The last sentence in the Introduction is modified:

*To further elaborate the connection between CK and glucose metabolism, this study hypothesized that CK is independently associated with glycated haemoglobin (HbA_1c_) in a population of nondiabetic subjects recruited from a general Causation population.*

Diabetes was defined as HbA1c ≥6.5%. Please justify why glucose and anti-diabetic medication were not used for diagnosis.

Reply:

This is explained in the methods:

*Those with diabetes (n = 1286) defined as HbA_1c_ ≥6.5% were excluded. This definition was used since fasting blood sugar and use of antidiabetic medication were not measured in the Tromsø study.*

Systolic and diastolic blood pressure should not be included in the same model. Their association parameters in Tables 3 and 4 are hard to explain due to the collinearity. Inclusion of systolic blood pressure is good enough.

Reply:

Diastolic blood pressure is taken out of the model in Table 3. Table 4 is deleted.

Best regards,

Svein Ivar Bekkelund

Corresponding author
